# Hydrothermal Synthesis of SnO_2_ Nanoneedle-Anchored NiO Microsphere and its Gas Sensing Performances

**DOI:** 10.3390/nano9071015

**Published:** 2019-07-15

**Authors:** Zhijie Wei, Qu Zhou, Jingxuan Wang, Zhaorui Lu, Lingna Xu, Wen Zeng

**Affiliations:** 1 College of Engineering and Technology, Southwest University, Chongqing 400715, China; 2 Electrical and Computer Engineering Department, Wayne State University, Detroit, MI 48202, USA; 3 College of Materials Science and Engineering, Chongqing University, Chongqing 400044, China

**Keywords:** hydrothermal synthesis, semiconductor, SnO_2_/NiO nanocomposite, NO_2_, performances, sensing mechanism

## Abstract

In this study, we reported a successful synthesis of a nanocomposite based on SnO_2_ nanoneedles anchored to NiO microsphere by a simple two-step hydrothermal route. The results show that the SnO_2_/NiO nanocomposite-based sensor exhibits more prominent performances than the pristine NiO microsphere to NO_2_ such as larger responses and more outstanding repeatability. The improved properties are mainly attributed to the p–n heterojunctions formed at the SnO_2_–NiO interface, leading to the change of potential barrier height and the enlargement of the depletion layer. Besides, the novel and unique nanostructure provides large and effective areas for the surface reaction. In addition, a plausible growth mechanism and the enhanced sensing mechanism were proposed to further discuss the special nanostructure which will benefit the exploration of high-performance sensors.

## 1. Introduction

Metal-oxide semiconductor sensor, which plays an important role among the most necessary devices in our everyday life, has been extensively applied in various areas of gas analysis such as the identification of hazardous gases, the monitoring of air quality and the detection of environmental pollution [1,2,3,4,5]. So far, diverse metal oxides, for instance TiO_2_ [6,7], ZnO [8,9], WO_3_ [10,11], NiO [12,13], and SnO_2_ [14,15], have received a lot of attention and been widely investigated. Among them, as a prominent p-type oxide semiconductor with a wide band-gap (3.6–4.0 eV), NiO has captured a tremendous amount of attention for the capability of gases detection due to its distinctive electronic nanostructure and excellent thermal stability [16,17]. However, traditional pristine NiO-based sensors have limited gas-sensing performances such as high working temperature, low sensitivity and unsatisfactory repeatability, which may impose restrictions on the fabrication of high-performance sensors [18]. In this regard, considerable efforts have been devoted to breaking through this limitation like morphology control [19,20], metal doping [21,22], nanocompositing [23,24] and so on. Off all the strategies, the synthesis of nanocomposites has been regarded as a desirable approach to substantially improve the performances of gas sensors [25]. Generally, for gas sensors, the route is expected to achieve the change of the nanostructure for more sufficient surface adsorption and the formation of a p–n heterojunction for more efficient electron transport. For example, Sun and his co-authors reported a facile hydrothermal synthesis of TiO_2_ nanorods decorated with NiO nanopartieles, and the sensor based on the materials showed ultrahigh sensitivity towards 200 ppm acetone in comparison to that of the pure NiO [26]. Zhu et al. synthesized hierarchical NiO/ZnO nanoflower, and the ethanol sensor based on the nanocomposite presented large gas responses and prominent repeatability due to the formation of the p–n heterojunction [27].

SnO_2_ which is one of the most important oxygen-deficient n-type metal oxides that has taken a dominant position in the area of gas sensing nanomaterials owing to its excellent chemical stability [28,29]. Besides, it is generally known that SnO_2_ has been proved to be a suitable oxide to form p–n or n–n heterojunction through the combination of other nanomaterials due to its small size effect [30]. Given this, the combination of SnO_2_ and NiO has been considered to be an effective approach to enhance the properties [31,32]. Kim et al. successfully synthesized SnO_2_–NiO composite nanoweb by a facile electrospinning route, and the fabricated sensor exhibited high response to NO_2_ and C_6_H_6_ which benefited from the presence of the p–n heterojunction [33]. Meng et al. found that NiO–SnO_2_ nanocomposite-based sensor exhibited more excellent gas-sensing performances than that of the pristine SnO_2_-based sensor, including larger gas responses, lower working temperature, shorter response/recovery times and so on [34]. They believed that the enhancement was caused by the catalytic effect of NiO and the presence of a p–n heterojunction. Hitherto, although diverse SnO_2_–NiO heterostructures have been synthesized with fascinating properties as mentioned above, to the best of our knowledge, nanocomposites based on a SnO_2_ nanoneedle-anchored NiO microsphere for gas sensing application have rarely been reported. Hence, the design and synthesis of a novel and unique composite nanostructure is still a challenging task to enhance the sensing properties of sensors.

Herein, the SnO_2_ nanoneedle-anchored NiO microsphere was triumphantly prepared by a simple two-step hydrothermal method. In the typical synthesis of SnO_2_/NiO nanocomposite, the pristine NiO microsphere was firstly synthesized and the nanocomposite was obtained based on the as-synthesized microsphere. The sensors fabricated with the SnO_2_/NiO nanocomposite presented higher gas responses and more prominent repeatability towards NO_2_ in comparison to the pristine NiO microsphere, further demonstrating that the improvement of the gas-sensing performances is closely related to the change of the pristine nanostructure and formation of the p–n heterojunction. Besides, the long-term stability of the sensors was measured over a period of 30 days which shows that the fabricated sensors exhibit excellent stability. In addition, we also proposed a possible growth mechanism of the novel nanostructure and a possible mechanism to explain the improved sensing properties. 

## 2. Materials and Methods

### 2.1. Preparation of SnO_2_ Nanoneedle-anchored NiO Microsphere

All involved chemicals used to synthesize the SnO_2_/NiO nanocomposite were analytical-grade grade and were obtained from Chongqing, Chuandong Chemical Reagent Co., Ltd. China and were used directly without further purification. The pristine NiO microsphere and SnO_2_/NiO nanocomposite were synthesized hydrothermally.

To prepare the pristine NiO microsphere, 2 mmol NiCl_2_·6H_2_O was added into 30 mL deionized water and stirred in a beaker for 15 min. Subsequently, 25% NH_3_·H_2_O was added dropwise, adjusting the pH to about 10. Then, the mixed solution was transferred into an autoclave with a capacity of 50 mL, heated at 180 °C for 6 h. After the autoclave cooled down to room temperature, the precipitate was collected by centrifugation and then washed with deionized water and ethanol several times. The sample was dried at 60 °C overnight and then collected for characterization and subsequent hydrothermal treatment.

Furthermore, the SnO_2_/NiO nanocomposite was obtained based on the sample as-synthesized above. In a typical process, the as-obtained precipitate and 0.8 mmol SnCl_4_·5H_2_O were dissolved into 30 ml deionized water under intense magnetic stirring for 15 min. Then, 0.6 mL ethylenediamine (EDA) and several drops of NH_3_·H_2_O were added into the solution. After stirring for 20 min, the mixed solution was transferred into the autoclave, sealed and kept at 180 °C for 10 h. Cooling naturally, the precipitate was collected in the same route. Finally, the product was obtained via calcinating at 400 °C for 2 h.

### 2.2. Characterization

The crystallographic information of the products was collected by X-ray diffraction (XRD, Rigaku D/Max-2500 (Rigaku, Japan), Cu-Kα radiation with *λ* = 1.54 nm) over the 2θ range of 20°–85°. The surface morphologies of the pristine oxide and composite were observed via scanning electron microscopy (SEM, JSM-7800F, JEOL, Japan). Energy dispersive spectroscopy (EDS) analysis was examined with INCA 250 (Oxford, UK). Moreover, the chemical states of the as-prepared composite were analyzed via X-ray photoelectron spectroscopy (XPS, ESCALAB-MK, VG, UK) with Al-Kα as the excitation source.

### 2.3. Fabrication and Measurement of Gas Sensors

**Figure 1:**
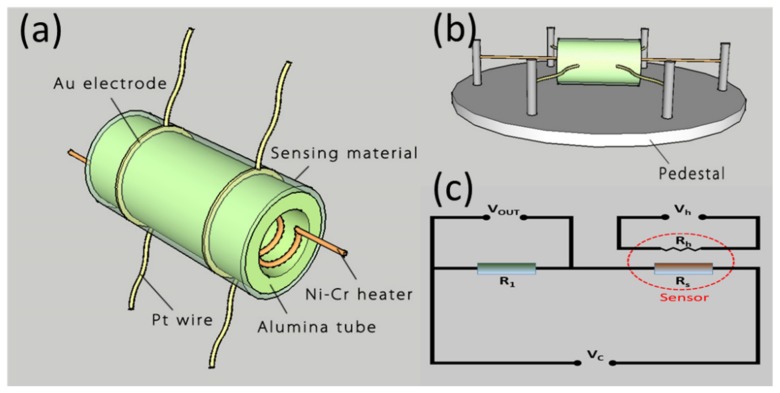
(**a**,**b**) Schematic illustration of the fabricated sensor. (**c**) Electric circuit of sensor device.

The gas sensor was designed with a side-heated structure (Figure 1a). Concretely, the as-prepared powder was dispersed in the water–ethanol mixed solution to form a homogeneous slurry which was coated onto an alumina ceramic tube evenly to form a sensing film. Afterwards, a pair of Au electrodes, which were connected with two Pt wires, were installed at every end of the tube. In addition, a Ni–Cr wire was inserted into the tube to change the heating current for the adjustment of the operating temperature, as shown in Figure 1b. At last, the fabricated sensor was aged at 300 °C for 2 weeks to ensure its high stability [35,36].

The gas-sensing properties to NO_2_ were measured on a Chemical Gas Sensor-8 gas-sensing analysis system (Beijing Elite Tech Co., Ltd., Beijing, China) controlled by a central computer. As presented in Figure 1c, the working temperature of the fabricated sensor was adjusted by changing the heating voltage (V_h_). The resistance of the tested sensor was calculated with the value of the output voltage (V_out_). The sensor was put into the airtight chamber with air, and then the concentration of the target gas was controlled by the injection of a certain amount of the gas. The carrier gas of the gas-sensing analysis system is O_2_, and the volume of the gas chamber is 20 L. Besides, the flux of the target gas and carrier gas is 20 ml/min. In this paper, the gas response of the fabricated sensor is defined as the ratio R_g_/R_a_, where R_g_ is the electrical resistance of the sensor in target gas and Ra the value in air [37]. 

## 3. Results and Discussion

### 3.1. Structural and Morphological Analysis

**Figure 2:**
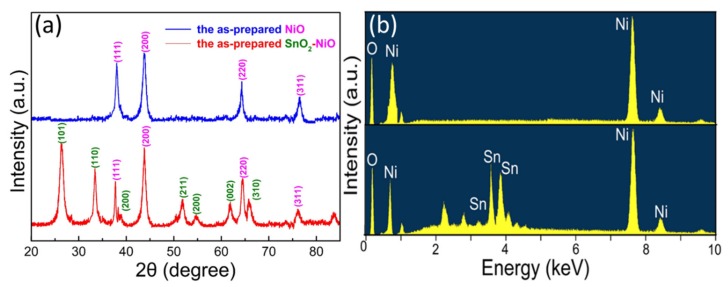
(**a**) XRD pattern of the synthesized samples. (**b**) EDS spectrum of the SnO_2_/NiO nanocomposite.

As demonstrated in Figure 2a, the XRD pattern was used to investigate the phase and crystalline structure of the obtained samples. From the typical pattern, it is obvious that the characteristic peaks at 36.9°, 43.2°, 63.1° and 75.4° could be assigned to the planes of (111), (200), (220) and (311), perfectly indexing to the NiO (JCPDS 78-0423). To confirm the synthesis of the composite, it is obvious from another result that the diffraction peaks around 26.7°, 33.9°, 52.6°, and 62.0° could be indexed to (110), (101), (211), and (002) planes of the SnO_2_ (JCPDS 41-1445), verifying the coexistence of SnO_2_ and NiO. In addition, the high purity of the synthesized sample can be confirmed by all the sharp and clear diffraction peaks. Besides, to further observe the elemental composition of the samples, EDS analysis was carried out as seen in Figure 2b. The results indicate that the pristine sample contains O, and Ni elements and the nanocomposite contain O, Ni and Sn elements. 

**Figure 3:**
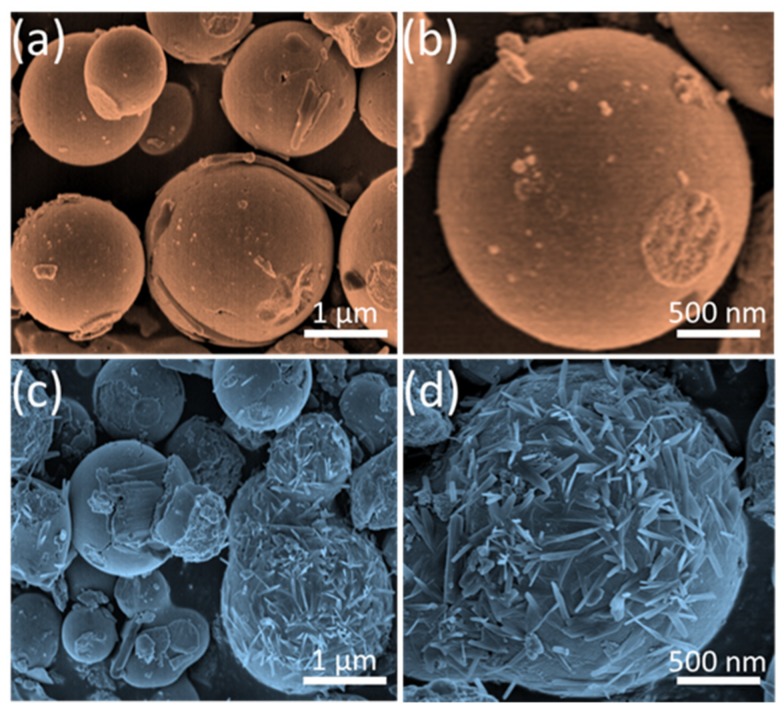
(**a**,**b**) SEM micrographs of the pristine NiO. (**c**,**d**) SEM micrographs of the SnO_2_/NiO nanocomposite.

The surface morphologies of the pristine NiO and SnO_2_/NiO nanocomposite were characterized by SEM observations. As displayed in Figure 3a,c, both the samples demonstrated a sphere-like morphology with relatively regular shapes. Higher magnification SEM micrographs of the two single microspheres are illustrated in Figure 3b,d. From Figure 3b, it can be observed clearly that the microsphere has a smooth surface morphology with a uniform diameter of 2 μm. The detailed structure of the nanocomposite is demonstrated in Figure 3d and the result shows that the microsphere with the same diameter of 2 μm was anchored by numerous nanoneedles on the surface, making the morphology rough and loose which provides large and effective areas for the surface reaction.

**Figure 4:**
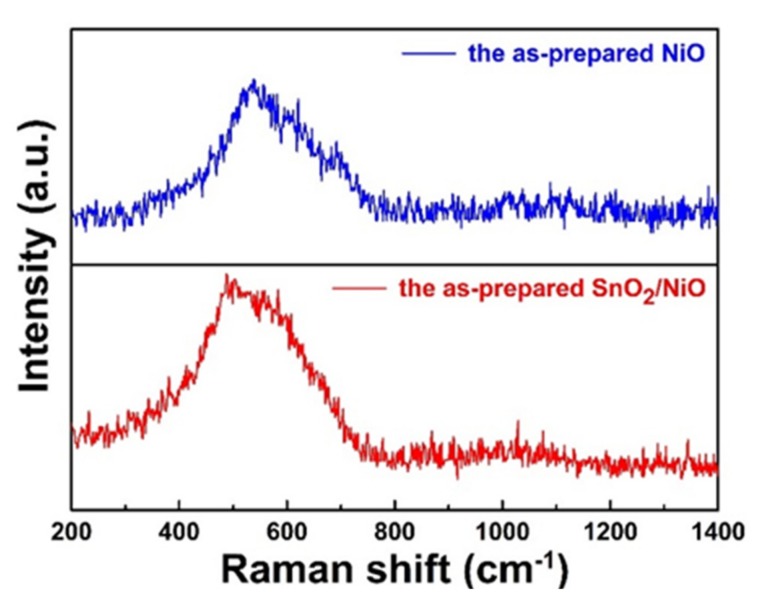
Raman scattering spectra of the pristine NiO and SnO_2_/NiO nanocomposite.

Raman scattering was used to confirm the synthesis of NiO and study the structural transition. As shown in Figure 4, the Raman spectra of the pristine NiO and the SnO_2_/NiO nanocomposite were carried out from 200 to 1400 cm^−1^. It can be found from the pristine product that the strong and broad peak at 520 cm^−1^ can be attributed to the Ni–O stretching mode. It is generally known that the lattice distortion has an effect on the lattice parameters and cell volume, leading to the change of the Raman frequencies [38]. The results of the composite spectrum exhibited that the shift to the lower frequency side of the mode, which was caused by the introduction of Sn, suggested the synthesis of the SnO_2_/NiO nanocomposite.

**Figure 5:**
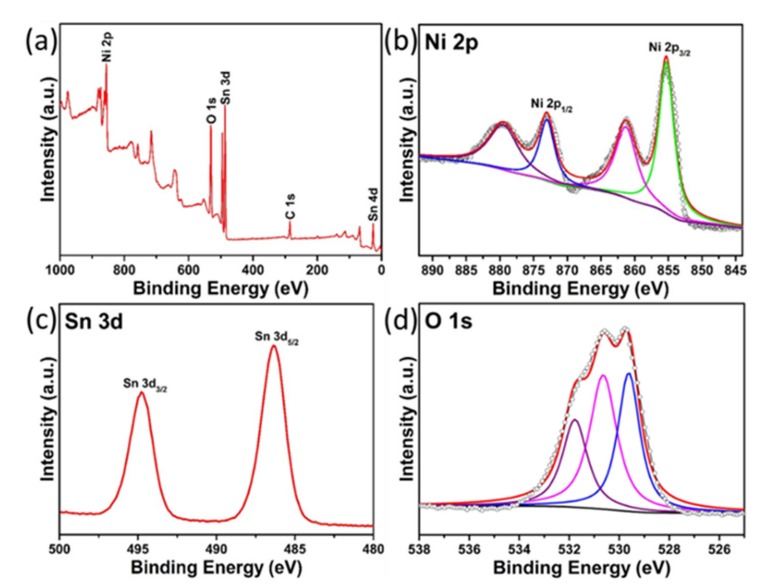
XPS spectra of the SnO_2_/NiO nanocomposite: (**a**) survey spectrum; (**b**) Ni 2p; (**c**) Sn 3d; (**d**) O 1s.

To further investigate the chemical states and surface chemical compositions of the SnO_2_/NiO nanocomposite, XPS analysis was performed as shown in Figure 5. From the full-wide (0–1000 eV) scanned survey spectrum (Figure 5a), it is obvious that the presence of Ni, O, Sn, and C in the SnO_2_/NiO nanocomposite, verifying again the high-purity of the as-synthesized composite. The Ni 2p XPS spectrum can be decomposed into four peaks in Figure 5b. The peaks located at 872.7 eV and 879.6 eV can be assigned to the Ni 2p_1/2_ and its satellite, while the peaks at 855.2 eV and 861.1 eV are ascribed to Ni 2p_3/2_ and its satellite, respectively. The observed four peaks can be attributed to the Ni^2+^, suggesting the presence of NiO. Figure 5c displays the high-resolution Sn 3d spectrum, from which we can find that the two measured strong peaks are located at 495.1 eV and 486.6 eV, which can be assigned to the Sn 3d_3/2_ and Sn 3d_5/2_, respectively. The two peaks and their spin-orbit splitting (8.5 eV) indicate the presence of Sn^4+^ in SnO_2_. The high resolution O 1s spectrum can be deconvoluted into three peaks as shown in Figure 5d. These peaks at 529.7 eV, 530.8 eV and 532.0 eV matched well with three different O species of the composite. The O 1s peaks at 529.7 eV are affected by the defect of the crystal, and the middle binding energy (530.8 eV) is ascribed to the lattice oxygen of SnO_2_/NiO nanocomposite, while the high binding energy (532.0 eV) can be assigned to the absorbed oxygen ions on the surface [34,39,40].

### 3.2. Growth Mechanism

**Figure 6:**
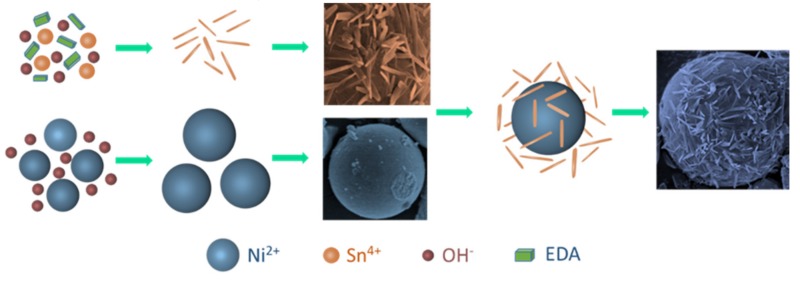
Schematic diagram of the possible growth mechanism of the SnO_2_/NiO nanocomposite.

Based on the analysis and experimental observations, a plausible growth process of the SnO_2_ nanoneedle-anchored NiO microsphere was proposed, as sketched in Figure 6. The smooth NiO microsphere was formed through the first hydrothermal treatment. First, Ni(OH)_2_ nucleus was formed as a precursor, owing to Ni^2+^ reacting with OH^−^ at the initial stage of the heating process. Then, the pristine microsphere was formed with the self-assembly and had limited growth. To synthesize the SnO_2_ nanoneedle-anchored NiO microsphere, EDA was added in the second hydrothermal treatment as a surfactant and nanostructure-directing agent. Specifically, the Sn(OH)_4_ nucleus was developed for numerous single rods owing to the oriented effect of EDA. With the heating time increasing, the pristine rods self-aggregated to eliminate the surface energy and then formed the nanoneedles. Consequently, through continuous aggregation with the assistance of EDA and subsequent calcination, the SnO_2_ nanoneedle-anchored NiO microsphere was obtained.

### 3.3. Gas Sensing Properties

To investigate the influence of the gas-sensing performances caused by the nanocompositing, the gas sensors were fabricated and then the NO_2_ sensing performances were measured. Firstly, the fabricated sensors based on the pristine NiO and the SnO_2_/NiO nanocomposite were exposed to 20 ppm NO_2_ at different temperatures (110–410 °C) to find out the optimum working temperature.

**Figure 7:**
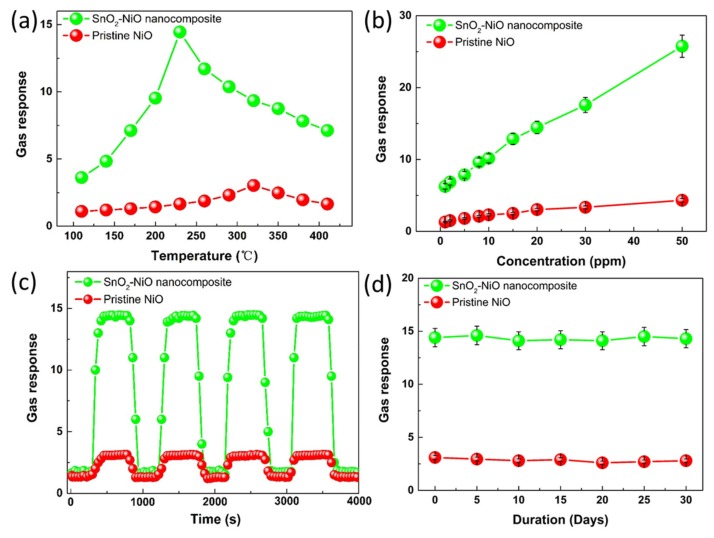
(**a**) The gas responses of the two samples to 20 ppm NO_2_ at various working temperatures. (**b**) The gas responses of the two samples towards various concentration of NO_2_ at the optimum temperature of 230 °C. (**c**) The dynamic responses of the two samples at the optimum temperature of 230 °C under 20 ppm NO_2_. (**d**) The stability of the two samples towards 20 ppm NO_2_ at the optimum temperature of 230 °C.

As shown in Figure 7a, the gas responses of the two sensors increased with the increasing working temperature to a certain value and then decreased with further increasing temperature. The behaviors of the temperature characteristic may be ascribed to kinetics and thermodynamics. At a lower temperature region, the low responses are caused by the high activation energy of the target gas adsorption. Besides, the active oxygen species on the material surface are limited owing to the low activation energy which can cause the transfer of electrons. With the working temperature increase, the oxygen and target gas adsorbed effectively, leading to the accelerated reaction between the active oxygen species and adsorbed target gases. During the increased temperature process, the response reaches the maximum with a certain temperature and the condition may be regarded as a balance of the adsorption and desorption. When the working temperature further increased, the balance was broken and the responses were decreased due to the increased desorption rate, resulting in the escape of target gases before effectively adsorbing on the material surface. In this work, the more obvious behavior and higher responses of the SnO_2_/NiO nanocomposite can be observed, and the result can be attributed to its rough and loose morphology which provides large and effective areas for the surface reaction. It is worth noting that the optimum working temperatures of the two samples are 320 °C and 230 °C, whose corresponding responses are 3.03 and 14.45, respectively.

Then, Figure 7b depicts the gas responses of the two sensors towards different concentrations of NO_2_ (1–50 ppm) at their own optimum temperature. It can be found that the responses of the two sensors increased obviously linearly with the increasing NO_2_ concentration in the tested range. In addition, it is also worth noting that the responses of the sensor based on the SnO_2_/NiO nanocomposite exhibit a more excellent performance than the pristine NiO.

Moreover, the response–recover curves towards 20 ppm NO_2_ at the optimum working temperatures were measured to ensure the repeatability. It can be seen from the result (Figure 7c) that the response of the two sensors goes up rapidly when NO_2_ is in but returns to the initial condition when the gas is out. The result shows that the fabricated sensors have excellent repeatability after four reversible cycles. The measured gas-sensing performances verify again that the sensor based on the nanocomposite shows higher responses than the pristine NiO. The response and recovery characteristics of the two sensors further demonstrate that the nanocomposite based on SnO_2_ nanoneedles anchored to the NiO microsphere is relatively more sensitive to NO_2_ than the NiO microsphere. As discussed above, the SnO_2_/NiO nanocomposite may be a potential material for sensing application as compared to the pristine NiO.

To confirm the long-term stability of the NO_2_ sensors, further responses were measured over a period of 30 days, as displayed in Figure 7d. The gas-sensing properties of the two sensors to 20 ppm NO_2_ at their own optimum temperature were tested every 5 days. The result shows that the fabricated sensors exhibit excellent stability with inapparent drift of responses.

Besides, NO_2_ sensing characteristics of the SnO_2_/NiO nanocomposite compared with some reported nanomaterials are listed in Table 1, from which we can find that the obtained SnO_2_/NiO nanocomposite in the present work shows more excellent NO_2_ responses than those reported papers because of the change of nanostructure and the formation of the p–n heterojunction.

### 3.4. Gas Sensing Mechanism

It is known that the change of electric resistance caused by the reaction between adsorbed oxygen species and tested gases are used to demonstrate the basic gas-sensing mechanism [46]. Before the injection of NO_2_, the oxygen molecules have adsorbed on the materials because of their strong electronegativity. During the process, the electrons in the conduction band were captured and then the oxygen molecules were reduced to chemisorbed oxygen ions which are O^2−^ (below 100 °C), O^−^ (100–400 °C) and O_2_^−^ (above 400 °C) [47]. Generally, when the electrons transfer from the oxides to the chemisorbed oxygen ions, the electron concentration of the oxides on the surface decreases obviously. For p-type NiO, the adsorption of oxygen molecules is promoted by the capturing of electrons from the valance band, leading to a holes accumulation layer and a low resistance in comparison to the core regions. On the contrary, n-type SnO_2_ shows a different behavior. Because of the transfer of conduction band electrons, a depletion layer can be formed on the surface of the mental oxides which will make the resistance increase. The oxygen chemisorption process can be described as follows [40]:

(1) $$O_{2}\left(gas\right)\to O_{2}\left(ads\right)$$

(2) $$O_{2}\left(ads\right)+2e^{-}↔2O^{-}\left(ads\right)$$

When NO_2_ molecules were injected, the oxidizing target molecules reacted with chemisorbed oxygen ions. With higher electron affinity compared with adsorbed oxygen, NO_2_ molecules extract more electrons, as shown in the following relations:

(3) $$NO_{2}\left(gas\right)\to NO_{2}\left(ads\right)$$

(4) $$2NO_{2}\left(ads\right)+2O^{-}\to 2NO_{2}{}^{-}+O_{2}\left(ads\right)$$

**Figure 8:**
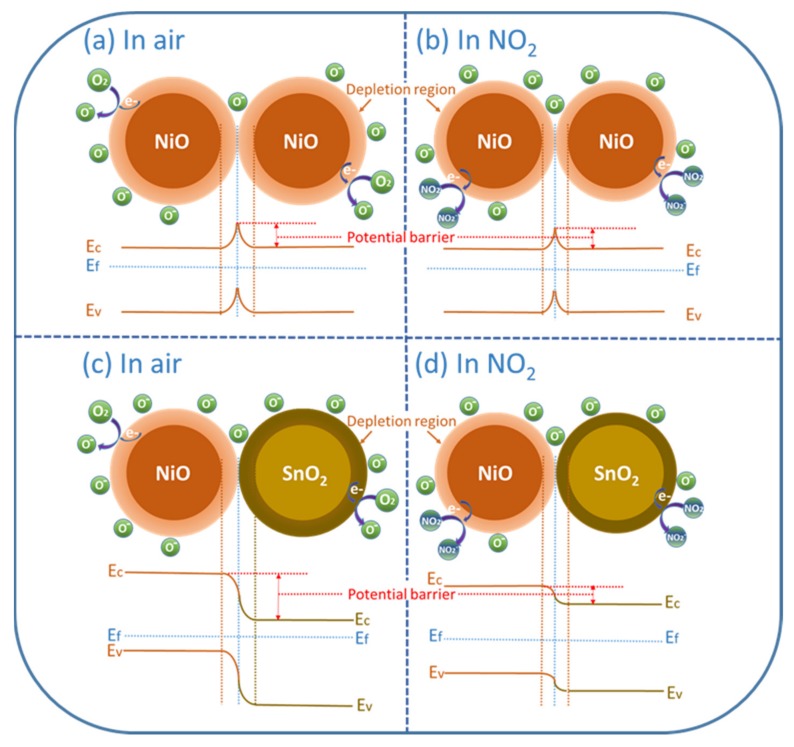
The sensing mechanism of (**a**,**b**) pristine NiO and (**c**,**d**) SnO_2_/NiO nanocomposite.

Besides, to further understand the enhanced properties caused by the formation of p–n heterojunctions, the sensing behaviors of pristine NiO and SnO_2_/NiO nanocomposite were investigated. As discussed above, when pristine NiO sensing materials are exposed to air, the oxygen molecules will adsorb on the surface and be ionized into O^−^, as depicted in Figure 8a. Figure 8b presents the sensing process and reaction of the pristine NiO-based sensor in NO_2_. In particular, in the case of the SnO_2_/NiO nanocomposite, the p–n heterojunctions are formed at the interface between the n-type SnO_2_ and n-type NiO with their different bandgap and Fermi Level which are caused by the charge carriers transport. The appearance of the heterojunctions cause the formation of the barrier and generate a depletion region. Specifically, as seen in Figure 8c, when the nanocomposite was exposed to air, the p–n heterojunctions made the fabricated sensor display a much higher electric resistance than the pristine sensor during the oxygen ionization process. When NO_2_ molecules were injected (Figure 8d), the target molecules reacted with adsorbed oxygen ions with a release of numerous trapped electrons, causing an obvious shrinkage of the p–n heterojunction depletion region [27,48]. Consequently, the obvious improved gas-sensing performances were observed due to the dramatic change of the sensor resistance.

**Figure 9:**
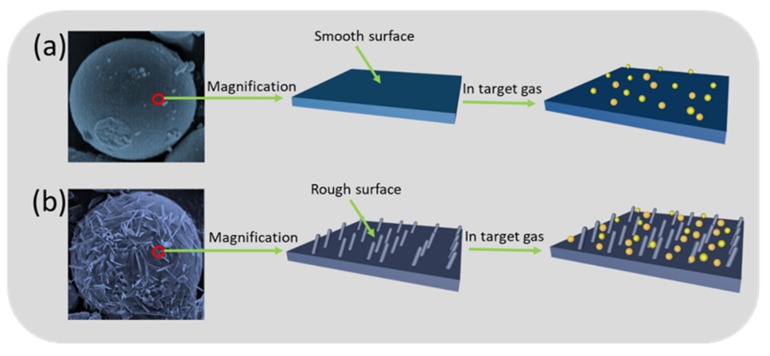
Schematic illustrations of the sensing mechanism. (**a**) The sensing mechanism of smooth surface. (**b**) The sensing mechanism of rough surface.

On the other hand, it is generally known that the surface morphology plays an important role in the gas-sensing process. In this work, the novel and unique structure of the SnO_2_/NiO nanocomposite was discussed in comparison to the pristine NiO. As shown in the SEM micrographs above, the result shows that the pristine NiO microsphere has a smooth surface while the nanocomposite has a rough surface based on SnO_2_ nanoneedles anchored to the NiO microsphere. More specifically, the NiO microsphere with a smooth surface possesses limited surface area for the oxygen and target gas molecules, indicating that the sensor exhibits lower response, as displayed in Figure 9a. On the contrary, the structure in Figure 9b possesses numerous nanoneedles on the surface, which provides numerous adsorption and desorption sites to the molecules. Besides, the rough surface increases the residence time of the molecules, leading to adequate surface reaction.

## 4. Conclusions

In summary, a novel nanocomposite based on SnO_2_ nanoneedles anchored to NiO nanosphere has been successfully obtained via a simple two-step hydrothermal method. The sensor fabricated with the SnO_2_–NiO nanocomposite exhibited prominent repeatability and much larger gas response (14.45) in comparison to the pristine NiO to 20 ppm NO_2_ at its optimum working temperature of 230 °C. The improved gas-sensing properties were discussed with a possible growth process and gas sensing mechanism, indicating that the enhancement was attributed to the change of nanostructure for more sufficient surface adsorption and the formation of a p–n heterojunction for more efficient electron transport.

## Figures and Tables

**Table 1:** Comparison of different NO_2_ sensors.

Materials	Concentration (ppm)	Operating Temperature (^o^C)	Response	Ref.
WO_3_ nanorods/graphene nanocomposites	1	300	5	[41]
Ppy–WO_3_ nanocomposites	100	R.T.	1.61	[42]
PTSA doped Ag-PPy nanocomposites	100	R.T.	1.68	[43]
Ni@ZnO/PANi nanocomposites	100	R.T.	1.75	[44]
WO_3_–In_2_O_3_ nanocomposites	1	140	2	[45]
SnO_2_/NiO nanocomposites	20	230	14.45	This work
